# Supplementary material for: Age-related delay in visual and auditory evoked responses is mediated by white- and grey-matter differences
Source: Nat Commun. 2017 Jun 9;8:15671. doi: 10.1038/ncomms15671 (PMC5472747; doi:10.1038/ncomms15671)
Supplement: Supplementary Information — Supplementary Figures and Supplementary Tables [file ncomms15671-s1.pdf]

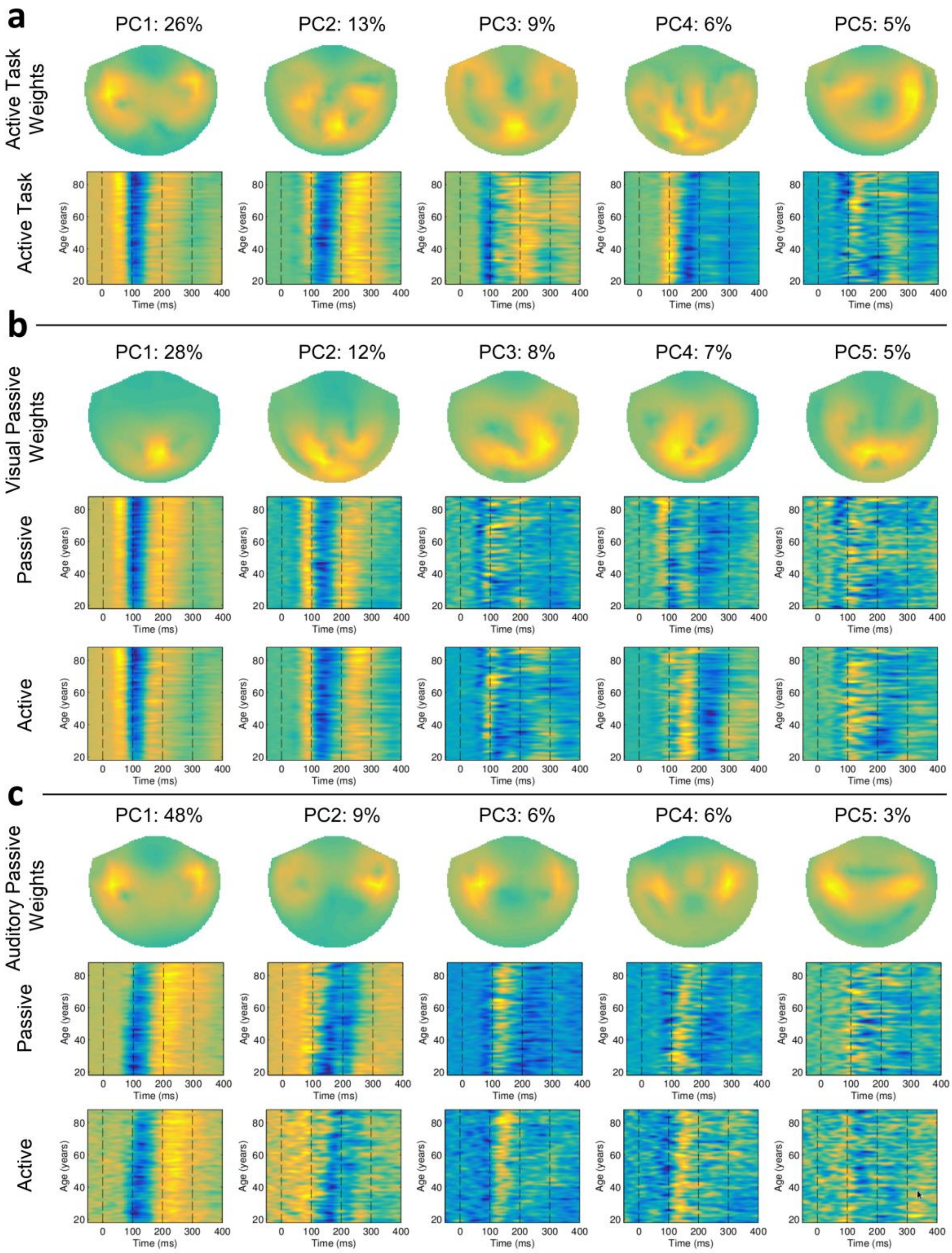

Supplementary Fig. 1: Principal component topography and time-series plots to show that using PCA on the active task data results in mixing of signals, and that this problem is alleviated in the passive session. **a**) Principal components from the active task. Auditory and visual components are not easily separable due to correlations in the evoked response timecourses. **b-c**) Principal component topographies from the visual and auditory conditions of the passive session. Timecourses are shown for both the passive and active task after applying sensor weights from the passive session (see row labels).

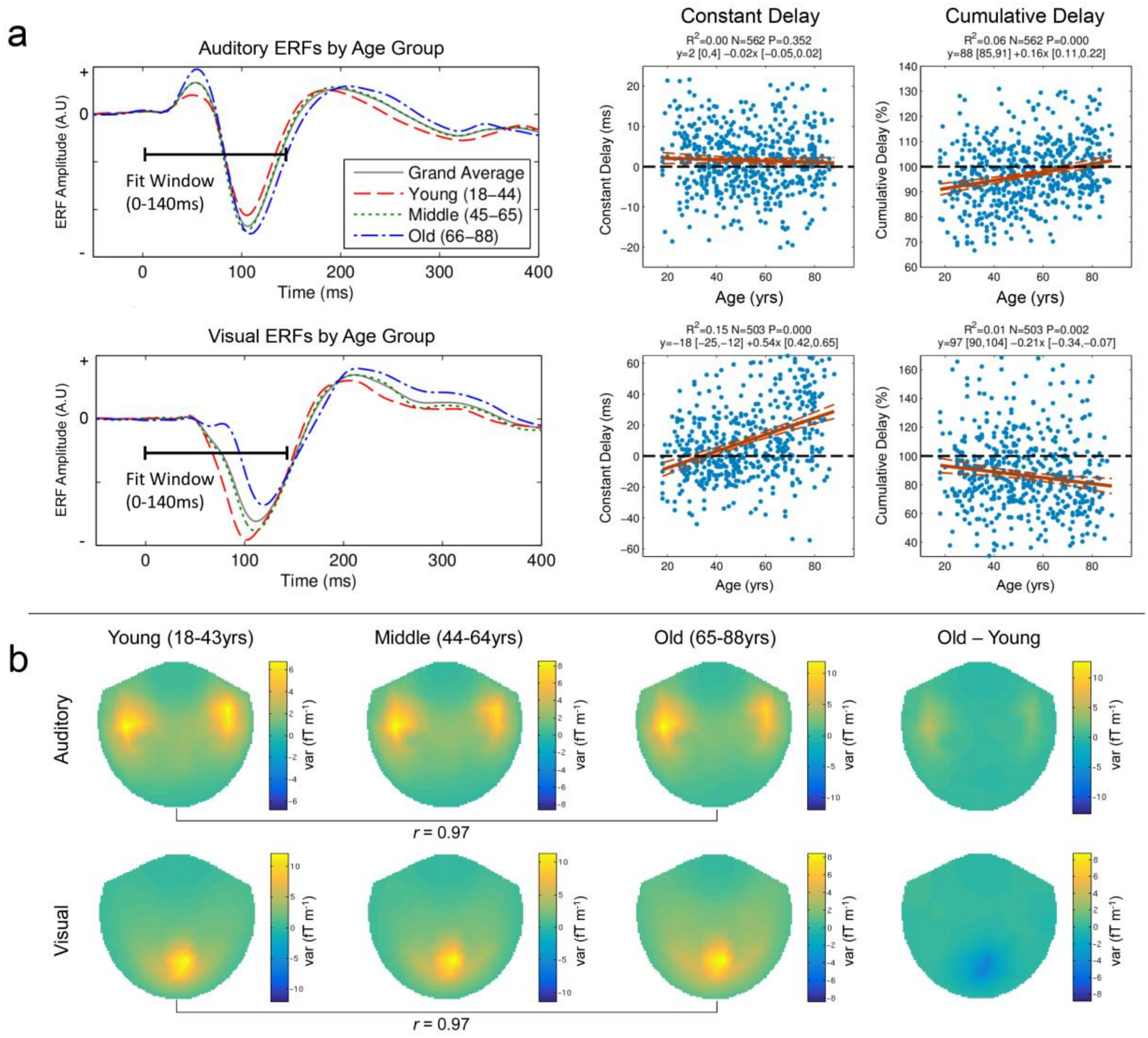

Supplementary Fig. 2. a) ERFs (first temporal component of PCA) are plotted for three equally-sized age groups (18-43, 44-64, 65-88yrs). No age-related delay is apparent in the P1m of the auditory ERF, while the N1m and P2m show increasing delay of their respective peaks. Our template fitting procedure makes the assumption that cumulative delay affects both early and late components. However, it is possible that a violation of this assumption (e.g., delay only affecting P2m, but not P1m or N1m) would result in spurious effects of age on cumulative delay. To test this possibility, we estimated delay parameters based on a shorter time window of 0-140ms (marked) to exclude late components (e.g., P2m) from the analysis. For both auditory and visual ERFs, the same dominant pattern, of age-related auditory cumulative delay, and age-related visual constant delay, was observed. b) 2D sensor plots showing the signal variance across sensors for each of the three age groups, along with a difference plot showing the difference between young and old groups. The old group has higher signal variance around auditory sensors for auditory stimuli, but lower variance around visual sensors for visual stimuli. However, there is negligible shift in the spatial distribution of variance, as indicated by the high correlation coefficients between young and old topographies.

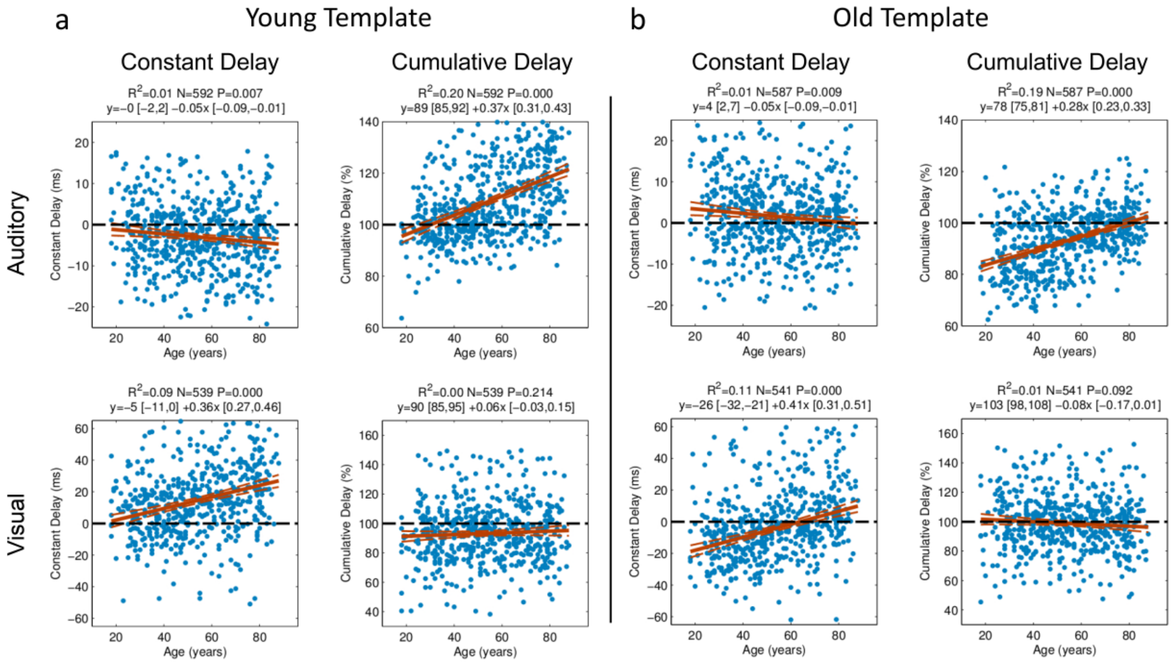

Supplementary Fig. 3. Effect of using different templates on the fitting procedure. As expected, using a template derived from young participants (< 30ys) resulted in a shift of the constant and cumulative delay estimates in the positive direction. Similarly, using a template from older subjects only (>60yrs) resulted in a negative shift of delay estimates. Nonetheless, the pattern of significant age effects was identical to that when using a template averaged across all participants, i.e., on visual constant delay and auditory cumulative delay.

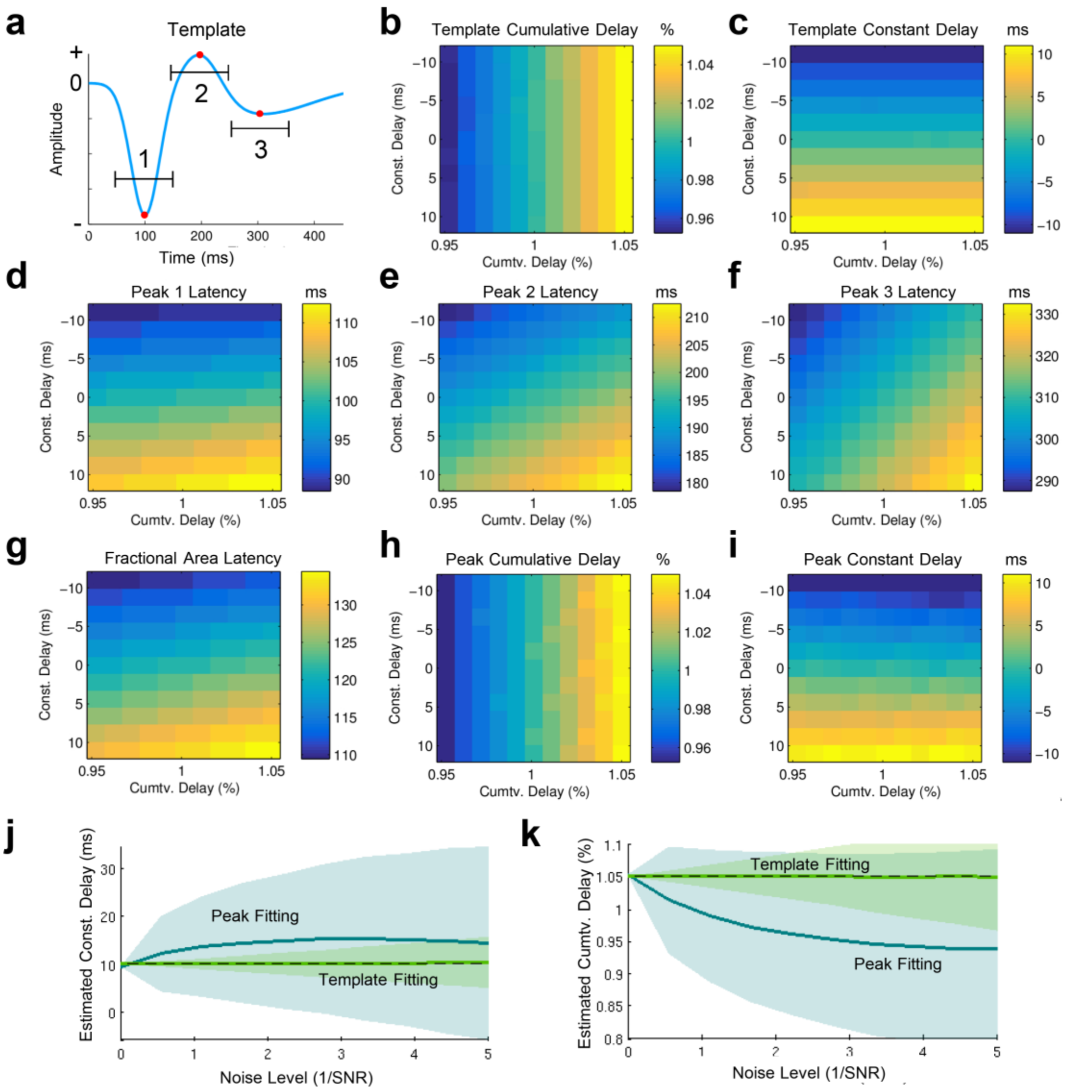

Supplementary Fig. 4: In order to compare the performance of the present template-fitting with previous peak-fitting approaches, we performed several tests on an idealised simulated evoked response (panel **a**). The simulated ERF was generated by adding together Gaussian peaks of varying parameters: amplitude (A), latency (L) and width (W) [peak 1: A = -1, L = 100ms, W = 25ms; peak 2: A = .5, L = 200ms, W = 35ms; peak 3: A = -1, L = 300ms, W = 100ms]. Peak fitting was achieved by defining three time windows (labelled in **a**) centred on each of the three peaks with a window of +/-50ms. To simulate our hypothesised age-effects, the ideal ERF was then resampled (using equation 1 from methods) with simulated constant delay (11 points ranging from -10 to +10ms) and cumulative delay (11 points ranging 0.95 to 1.05). For each combination of constant and cumulative delay, eight estimates of delay were displayed on 2D grids as follows: **b-c**) With template-fitting, the orthogonal gradients confirm that when assumptions about the delay types are met (i.e. that delay affects the whole epoch), and no noise is present in the data, constant and cumulative delay are perfectly separable. The values obtained are also accurate, so that the value in the grid at location [+10, 1.05] reads 10 in **b** and 1.05 in **c**. **d-f**) Peak latencies alone however are affected by both constant and cumulative delay, the influence of each depends on the time point under observation. **g**) Fractional area latency (FAL) was calculated by finding the point at which the integral of the absolute value of the timecourse reached half of the maximum of the total integral. FAL was also unable to distinguish between constant and cumulative delay. **h-i**) By fitting all three peak latencies with a linear function, one can derive

estimates of constant delay (intercept) and cumulative delay (slope, analogous to peak-to-peak latencies). This method is also accurate when no noise is present in the data, but performs worse than template-fitting in the presence of noise, as shown in (**j-k**), where delay parameters were fixed (const. = +10ms, cumtv. = +1.05) and the fitting procedure repeated 10,000 times with 10 levels of white noise with a variance ranging from 0 to 5 times the variance of the simulated timecourse (1/SNR). The mean of each batch of 10,000 tests is plotted as a solid line, with standard deviations plotted as semi-transparent area around the mean. It is clear that template fitting provides more accurate estimates of constant and cumulative delay, even in the presence of relatively high levels of noise.

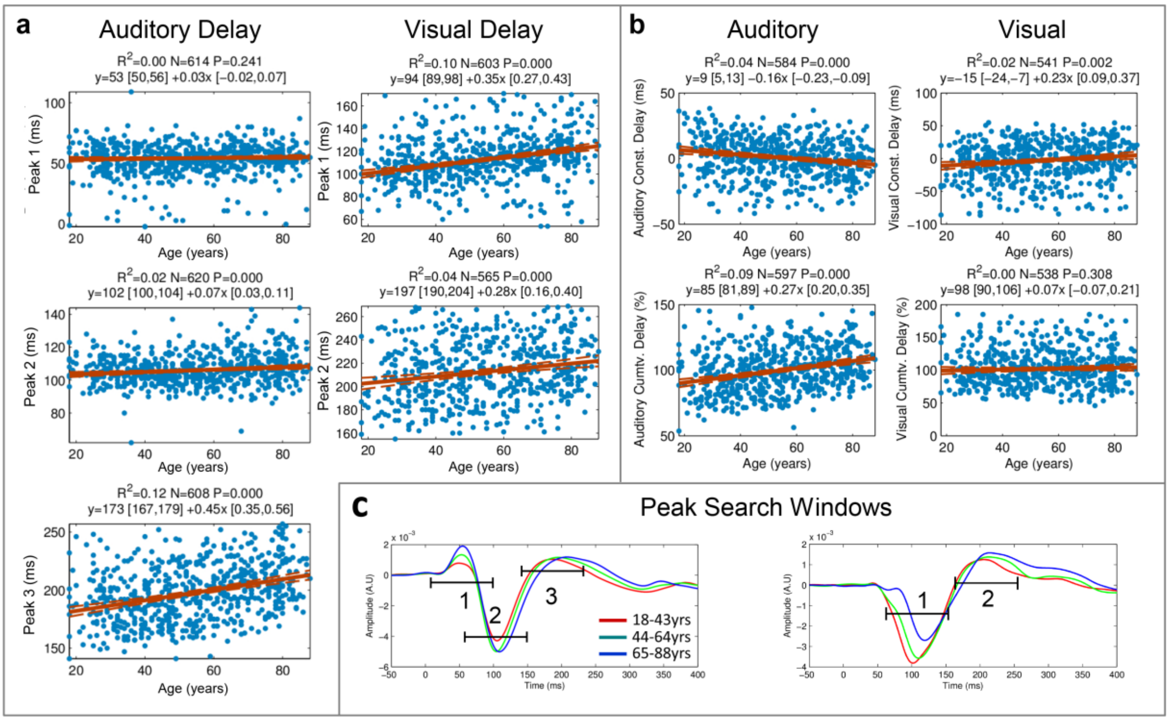

Supplementary Fig. 5: The same method used in Supplementary Fig. 4 was applied to the real data (though there were only two clear components in the visual case). **a**) Peak latencies for auditory and visual components. The effect of age on auditory peak delays is larger for later than earlier peaks, consistent with a cumulative delay (and with Iragui et al. 1993). Visual latencies reveal some age-related delay in peak 1 and 2: Although R^2 is lower in peak 2 (R^2=0.04), the slope of peak 1 (.35) is within the confidence bounds of peak 2 ([.16, .40]). **b**) As in Supplementary Fig. 4 (panels **h** and **i**), constant and cumulative delay were estimated by fitting a linear model to the peak data, and calculating the intercept and slope of those peaks, to estimate constant and cumulative delay respectively. Analysis of visual latencies revealed a similar pattern as the template-fitting approach, i.e, of visual constant delay in absence of visual cumulative delay. However, the variance explained by peak-fitting (R^2=0.02) was considerably lower than for template-fitting (R^2=0.11), suggesting that peak-fitting was not as sensitive. Analysis of auditory peak latencies revealed a positive effect of age on auditory cumulative delay (R^2=0.09), but a small negative effect of age on auditory constant delay (R^2=0.04). It is clear from examining plots in panel c that there is no negative relationship between age and constant delay in the auditory ERF (which would correspond to a negative shift of the P1m in the older subjects). This effect is likely a spurious result due to the lower sensitivity of peak fitting compared to template fitting approaches.

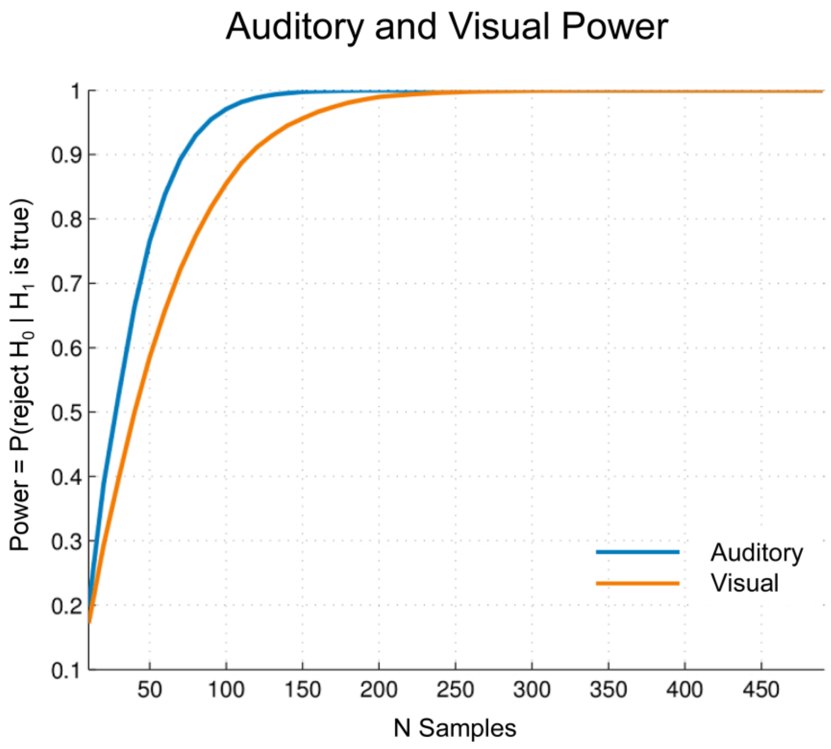

Supplementary Fig. 6: Non-parametric power calculations obtained by bootstrapped re-sampling, from N=10 to the full sample here, in steps of 10, with 10,000 re-samples per sub-group size. Blue and orange lines indicate the power (the probability of rejecting the null hypothesis at the $p<0.05$ level, given the alternative hypothesis is true). Therefore, P is calculated as the proportion of resamples in which the lower confidence interval was greater than 0.

Correlations Between Delay and Reaction Times

	Passive			***Active***		
	RT	*RT Cov = Age*	*N*	*RT*	*RT Cov = Age*	*N*
Age	--	--	--	0.06	--	609
Aud Const.	0.15***	0.15***	567	0.10*	0.12***	535
Aud Cumtv.	-0.02	-0.05	567	0.00	-0.03	535
Vis Const.	0.05	0.03	511	0.03	0.01	545
Vis Cumtv.	0.00	0.00	511	0.03	0.04	545

**P<0.05, **P<0.01, ***P<0.001*

Supplementary Table 1. Rank correlations (Spearman's Rho, r_s) of reaction times in the active task (RT) with age and delay estimates in both tasks (there were no RTs in passive task). Correlations were performed with and without age as a covariate. RT was not correlated with age (p<0.05), likely reflecting the non-speeded nature of the active task. There was a weak positive correlation between RT auditory constant delay in both passive and active tasks. No other delay parameters were correlated with RT.

Age-related Delay Estimates for Template and Peak-Fitting Methods

	Template Slope (ms/year) [CI]	***Peak Slope (ms/year) [CI]***
Auditory P1m	-.03 [-.06 .01]	.03 [-.02 .07]
Auditory N1m	.09 [.03 .15]	.07 [.03 0.11]
Auditory P2m	.32 [.21 .43]	.45 [.35 .56]
Visual N1m	.40 [.26 .53]	-.16 [-.23 -.09]
Visual P2m	.39 [.17 .61]	.27 [.20 .35]

Supplementary Table 2. Comparison of template vs. peak fitting (from Supplementary Fig. 5). Constant and cumulative delay estimates were converted to ms/year for a given peak using Equation 4 in Methods.
